# Derlin-like proteins are essential for the function of *Euglena gracilis* chloroplasts

**DOI:** 10.1093/plphys/kiag253

**Published:** 2026-04-29

**Authors:** Anežka Konupková, Zoltán Füssy, Priscila Peña-Diaz, Masami Nakazawa, Vladimír Hampl

**Affiliations:** Faculty of Science, Department of Parasitology, BIOCEV, Charles University, Vestec, Czech Republic; Scripps Institution of Oceanography, University of California San Diego, La Jolla, CA, United States; Faculty of Science, Department of Parasitology, BIOCEV, Charles University, Vestec, Czech Republic; Department of Applied Biological Chemistry, Graduate School of Agriculture, Osaka Metropolitan University, Osaka, Japan; Faculty of Science, Department of Parasitology, BIOCEV, Charles University, Vestec, Czech Republic

## Abstract

Despite the growing interest in basic research on *Euglena gracilis* and its importance in bioproduction, several key questions regarding its cell biology remain unanswered. One of them concerns the mechanism of protein import into its secondary chloroplasts, which are derived from a green alga. In this study, we have proved the essentiality of 2 rhomboid pseudoproteases, DerL-1 and DerL-2, which are predicted to localize in the membrane of the chloroplast envelope. We demonstrate that these proteins belong to a euglenid-specific subgroup unrelated to other eukaryotic rhomboid pseudoproteases. Silencing these proteins using RNA interference leads to a distinct phenotype marked by culture bleaching and delayed growth. This is accompanied by the loss of typical chloroplast structure and a reduction in the expression of a substantial portion of the chloroplast proteome, especially proteins involved in photosynthesis. We hypothesize that these pseudoproteases are involved in chloroplast protein import by forming a core component of the translocon across the middle envelope membrane. This indicates a remarkable case of molecular convergence, as other eukaryotes utilize rhomboid pseudoproteases—unrelated to euglenid proteins—for protein import into plastids derived from red algae.

## Teaser

Membrane pseudoproteases sustain chloroplast function in *Euglena* and reveal an unexpected protein import system, highlighting convergent evolution in cellular machinery.

## Introduction

*Euglena gracilis* chloroplasts originated approximately 650 to 540 million years ago via secondary endosymbiosis, in which a heterotrophic euglenid ancestor engulfed a green alga related to *Pyramimonas parkeae* (Turmel et al. 2009; Hrdá et al. 2012; Jackson et al. 2018). The chloroplasts are surrounded by a triple-membrane envelope (Lefort-Tran 1981), which exhibits a glycosyldiacylglycerol content suggesting that 2 membranes descended from the chloroplast of the green alga. The third, outermost, membrane thus likely derives from the plasma membrane of the green alga or the phagosome of the euglenid host (Tomečková et al. 2020). Most nucleus-encoded proteins imported into the chloroplast contain N-terminal multipartite targeting pre-sequences of 2 classes. Class I is composed of a hydrophobic N-terminal signal peptide (SP), followed by a transit peptide (TP) and a transmembrane stop-transfer sequence (STS), while class II lacks the STS (Durnford and Gray 2006; Novák Vanclová et al. 2020). These targeting pre-sequences were experimentally shown to be essential for import into *E. gracilis* chloroplasts in the presence of other key factors, Golgi, adenosine triphosphate (ATP), guanosine triphosphate (GTP), and light (Sláviková et al. 2005). The TP itself was proven to be sufficient for the import into pea chloroplasts (Sláviková et al. 2005).

Given the experimental data, the following protein import pathway was proposed. In the first step, nucleus-encoded proteins are co-translationally directed by the SP into the endoplasmic reticulum (Sulli et al. 1999) and then transported via vesicles through the Golgi apparatus (Sulli and Schwartzbach 1995, 1996) to the outermost membrane of the chloroplast. This process is not sensitive to N-ethylmaleimide, an inhibitor of SNARE-based fusion of transporting vesicles (Sláviková et al. 2005). Regardless, the presence of the SNARE protein GOSR1 and Rab5 GTPase in the proteome of the chloroplast fraction suggests they may be involved (Novák Vanclová et al. 2020). The translocation machinery across the 2 inner membranes of *Euglena* chloroplast, derived from the chloroplast of green alga, involves only a minimal set of components homologous to those in green algae and plants (Záhonová et al. 2018; Novák Vanclová et al. 2020). Primary chloroplasts of plants and green algae contain the TOC and TIC (translocons on the outer and inner chloroplast membranes) complexes, which form channels across these membranes. The TOC complex facilitates the translocation of proteins featuring an N-terminal TP sequence, recognized by specific receptors from the Toc34 and Toc159 protein families, through a Toc75 beta barrel protein forming a channel (Schleiff et al. 2003; Jin et al. 2022). For the TIC complex, 2 competing models for translocation have been proposed. The classical model features Tic110 as the main component, functioning with stromal chaperones as ATP-dependent import motors. A more recent model proposes that the TIC complex comprises a channel formed by Tic20, along with Tic214, Tic56, and Tic100, which interacts directly with the TOC complex (Soll and Schleiff 2004; Gutensohn et al. 2006; Kikuchi et al. 2009; Kovács-Bogdán et al. 2010, 2011; Nakai 2018; Jin et al. 2022). Searches through transcriptomic and proteomic data of *E. gracilis* and its closely related species, *E. longa*, revealed no homologs of TOC complex subunits, and out of TIC complex subunits, only 3 divergent homologs of Tic21 were identified (Záhonová et al. 2018; Novák Vanclová et al. 2020). Tic21 is structurally similar to Tic20, and in thale cress (*Arabidopsis thaliana*), both proteins participate in forming a channel in the inner membrane (Teng et al. 2006; Kikuchi et al. 2009; Kovács-Bogdán et al. 2011). Whether the 3 paralogues of Tic21 in *E. gracilis* fulfil this function remains to be elucidated. Finally, the protein import model into *E. gracilis* chloroplasts assumes that after reaching the chloroplast stroma, the TP of the translocated protein is cleaved by processing peptidases, and the mature protein either remains in the stroma or is imported into thylakoids. This import occurs through 1 of the 3 pathways—TAT, Alb3/SRP, or SEC. Components of all 3 pathways are present in *E. gracilis,* and they consist of TatA and TatC; 2 paralogs of Alb3; and SecA, SecE, and SecY, respectively (Záhonová et al. 2018; Novák Vanclová et al. 2020).

Due to the lack of identifiable homologs of TOC, there is no indication of how the proteins are translocated across the middle envelope membrane. However, chloroplast proteomics data contain 2 transmembrane proteins related to rhomboid pseudoproteases known as Derlin-like proteins (DerLs) (Novák Vanclová et al. 2020), which may form an alternative transport system. Rhomboid proteases are intramembrane serine proteases possessing a core of 6 transmembrane domains and an intramembrane active site containing a serine and histidine catalytic dyad. They play various roles, such as facilitating cell signaling in animals, quorum sensing in bacteria, maintaining mitochondrial homeostasis, and involvement in host invasion in unicellular parasites (Urban and Dickey 2011). Alternatively, Derlin pseudoproteases, which are their homologs lacking proteolytic activity, have taken on a different role in the ERAD (Endoplasmic Reticulum-Associated Degradation) pathway. In this pathway, Derlins function as pore-forming components of a membrane complex, facilitating the transport of ubiquitinated proteins from the ER lumen into the cytoplasm for degradation (Wu et al. 2020; Rao et al. 2023). In red algae-derived complex plastids of Cryptophyta, Alveolata, Stramenopiles, and Haptophyta, the ERAD machinery of the algal endosymbiont was transformed to SELMA (Symbiont-specific ERAD Like Machinery), now responsible for import of proteins across the second envelope membrane (Stork et al. 2012; Ponce-Toledo et al. 2025). The localization of Derlin proteins in the plastidial membrane was experimentally confirmed in stramenopiles and apicomplexans (Sommer et al. 2007; Agrawal et al. 2009; Hempel et al. 2009; Kalanon, Tonkin, and McFadden 2009), and the requirement for both Derlins and a functional ubiquitylation machinery in protein import has been demonstrated in the apicoplast of *Toxoplasma gondii* (Agrawal et al. 2009, 2013). On the other hand, the involvement of these proteins in the protein import into the secondary green alga-derived plastid of the chlorarachniophyte *Bigelowiella natans* is unlikely, since only 2 nucleus-encoded Derlin-like proteins were found in its genome, both localized in the endoplasmic reticulum (Hirakawa et al. 2012).

In this work, we demonstrate that the Derlin-like proteins detected in the proteome of *E. gracilis* chloroplasts are essential for their proper functioning. Silencing these proteins leads to impaired cell growth and a strong chloroplast phenotype manifested in bleaching, decreased expression of many chloroplast proteins, and a morphological reduction of these organelles. We suggest that Derlin-like proteins may have convergently evolved into components of the elusive translocon that transports proteins across the middle membrane of the chloroplast envelope of euglenids.

## Results

### Sequence analyses suggest the existence of a euglenid-specific clade of Derlin pseudoproteases

**Figure 1:**
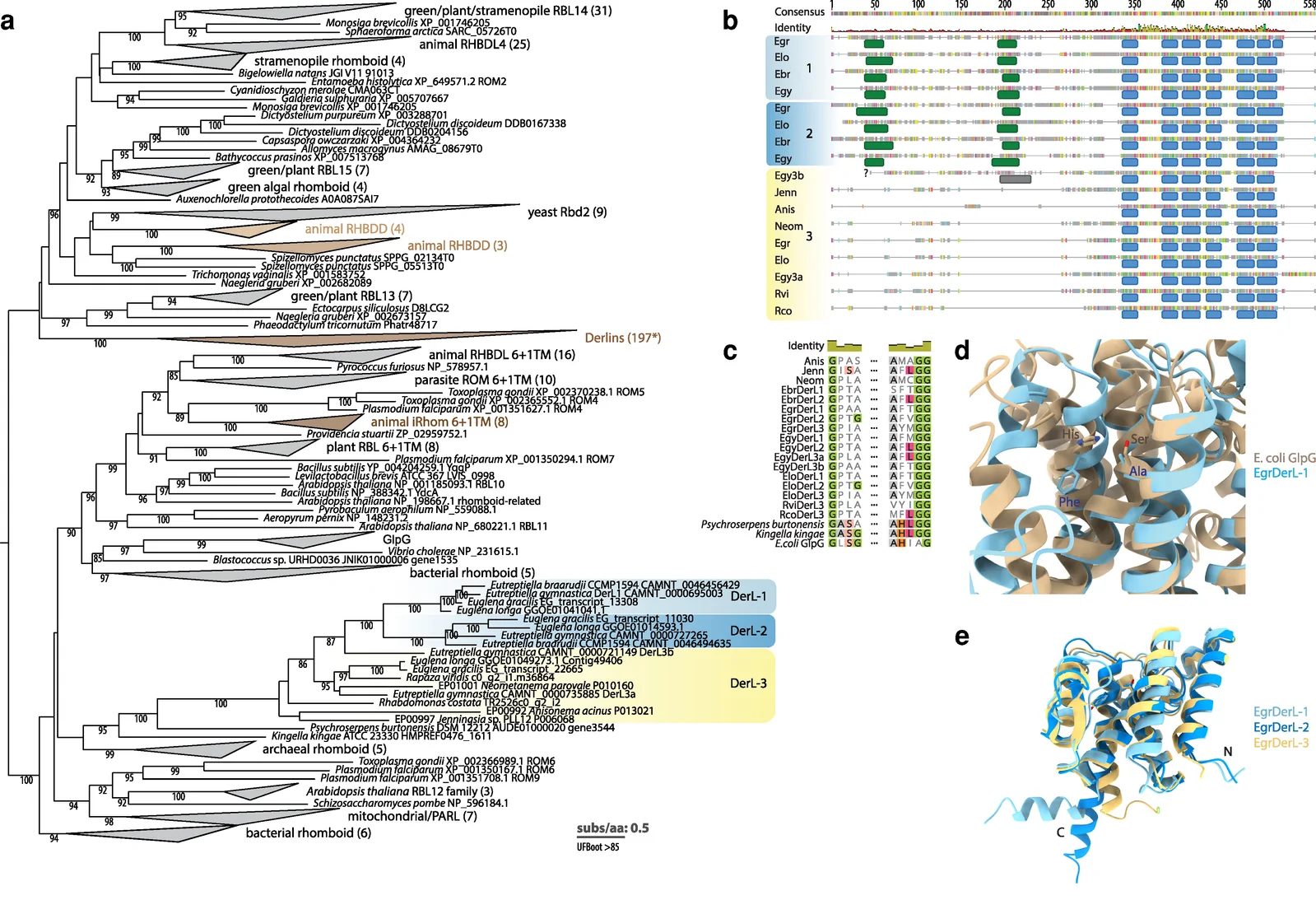
**Euglenid Derlin-like proteins evolved from prokaryotic sources and independently from other eukaryotic rhomboid superfamily proteins.** a) Phylogeny of updated reference rhomboid, rhomboid-like, and Derlin sequences with euglenid homologs branching in a sister position to bacterial and archaeal rhomboid proteases. The numbers in brackets indicate the number of sequences in collapsed clades; the clades in gray denote active proteases, those in color denote pseudoproteases. *Derlins include SELMA Derlin clades 1-1 and 1-2. The interactive version of the tree is available on iTOL (see Data availability). b) Schematic sequence alignment showing the conserved domain structure of the Derlin-like part of the euglenid homologs (in blue, structure inferred from AlphaFold 3 Server model alignments) and the differences in targeting pre-sequences that divide the family into 3 clades. DerL-1 and DerL-2 possess typical class I targeting pre-sequences, with transmembrane domains marked in green, which in *E. gracilis* localize the proteins to plastids, whereas DerL-3 displays a predicted mitochondrial targeting pre-sequence. ? denotes the incomplete N-terminus of Egy3b, where localization could not be ascertained. c) Partial sequence alignment of the rhomboid active site-homology region. The Ser-His dyad facing one another in a submerged pocket of true rhomboid proteases is missing in euglenid homologs. The comparison includes Euglenida sequences, their closest matches from *Kingella*, *Psychroserpens*, and *E. coli*. For the full alignment, see Data availability. d) Structural comparison of the proteolytic pocket in *E. coli* GlpG protease (PDB:2NRF) and *E. gracilis* DerL-1. The residues of the proteolytic dyad (Ser201, His254) are not conserved in Euglenida regardless of the positions in the tertiary structure. e) Structural alignment of the 3 mature *E. gracilis* Derlin-like proteins. The structures are matching overall, with most differences occurring in the C-terminal helix. The amino acids in the (inactive) proteolytic pocket of the proteins are shown. Source data for all panels are provided in an online repository (see Data availability).

Three paralogs of Derlin-like (DerL) proteins were detected in *E. gracilis* data, as well as in the datasets of other euglenids (Fig. 1). Homologs of DerL-1 and DerL-2 were found specifically in species of euglenids possessing permanent secondary chloroplasts. The third paralogue, DerL-3, was also present in *Rapaza viridis*, a species harboring plastids stolen from algal prey (kleptoplasts), as well as in heterotrophic euglenids without chloroplasts (Fig. 1a). All 3 DerL paralogs form a robust clade sister to bacterial and archaeal rhomboid proteases, unrelated to Derlins or rhomboid proteases of eukaryotes, including those involved in ERAD and SELMA pathways (Fig. 1a). The overall phylogeny of this protein family is difficult to resolve, likely due to deep homologies and a high percentage of hydrophobic residues in the alignment, as the proteins contain 6 transmembrane domains. Yet, multiple conserved functional clades were resolved (including iRhom, RHBDD, green algal/plant RBLs, Derlins), which is consistent with a previous study (Urban and Dickey 2011).

The sequence alignment of euglenid DerLs showed that the rhomboid domain structure of 6 transmembrane helices is overall conserved (Fig. 1b). Furthermore, all DerL-1 and DerL-2 paralogs possess a typical class I multipartite plastid-targeting pre-sequence, in which the first 2 transmembrane domains represent the SP and the STS, respectively, with the plastid TP located between them (Fig. 1b). In contrast, the N-terminus of DerL-3 contains a predicted mitochondrial pre-sequence.

The active site of rhomboid proteases displays a Ser-His dyad facing one another in a submerged pocket (Urban and Dickey 2011). Although euglenid homologs possess this region, they do not encode this proteolytic dyad (Fig. 1c). Instead, at the Ser site, they encode a nonpolar residue or a hydroxylated Thr (Ser in one case), whereas at the His site, they encode a bulky hydrophobic amino acid (Phe, Tyr, or Met). The comparison included euglenid sequences, their closest matches from *Kingella* and *Psychroserpens*, and an assortment of other homologs. For the full alignment, see Data availability. Consistently, while the structural comparison of the proteolytic pocket in *E. coli* GlpG protease (PDB:2NRF) and *E. gracilis* DerL-1 shows good superimposition of the tertiary structures, the residues of the proteolytic dyad are not conserved in Euglenida (Fig. 1d). The structural alignments of all 3 mature DerLs found in *E. gracilis* match very closely, with main differences in the C-terminal helix (Fig. 1e; TM-score by TM-align: DerL-1 vs. DerL-2: 0.854; DerL-1 and DerL-3: 0.851; DerL-2 and DerL-3: 0.881).

### Silencing of DerL-1 and DerL-2 leads to delayed growth and a bleached phenotype

**Figure 2:**
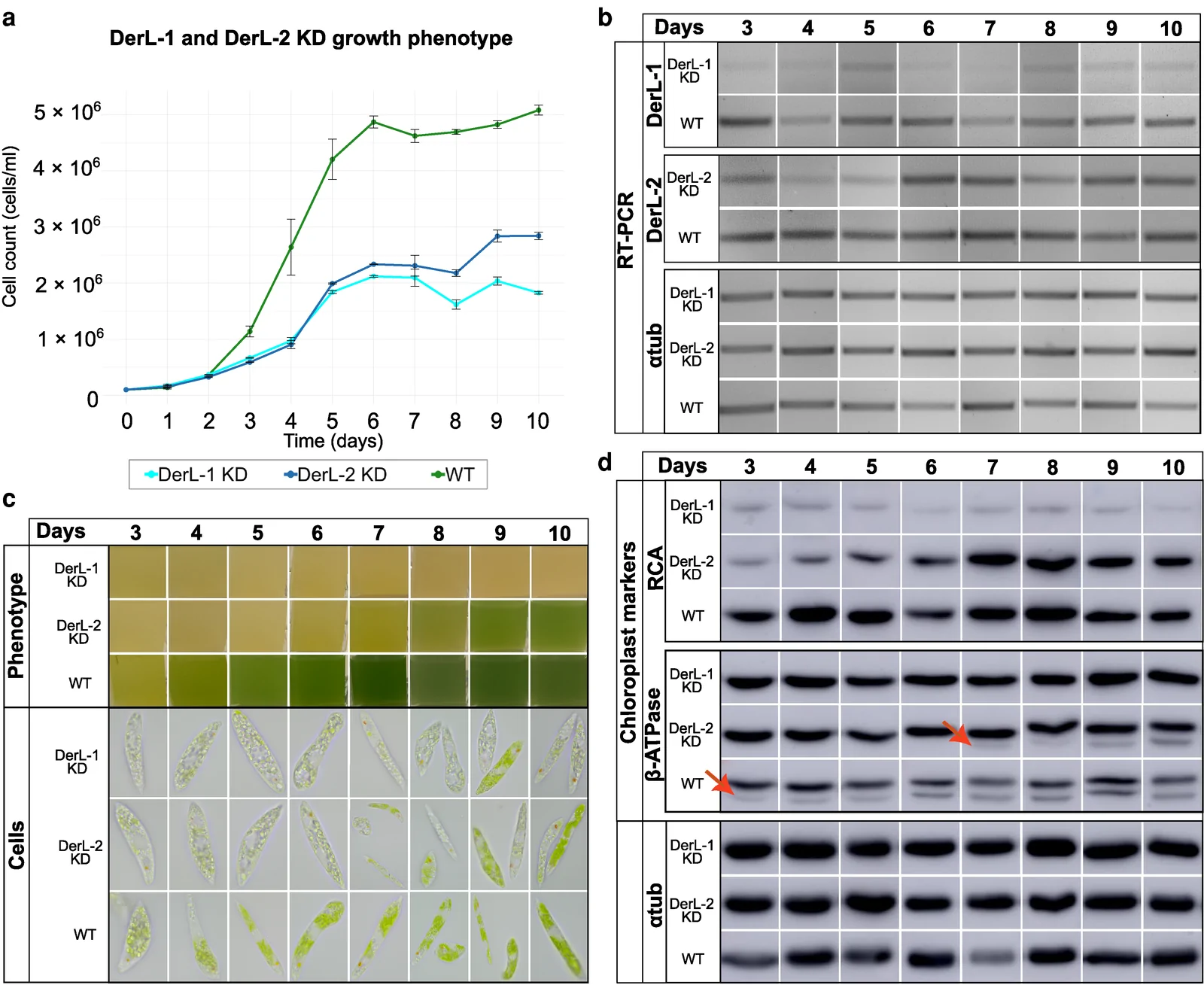
**Silencing of DerL-1 and DerL-2 leads to a growth delay, bleached phenotype and suppression of chloroplast marker expression.** a) Downregulation of DerL-1 and DerL-2 causes a growth delay in the cells as compared to wild type (WT) when observed for 10 d of growth. The error bars represent SD (*N* = 3). b) RT-PCR shows the stable silencing effect with reduced DerL-1 gene expression during the whole 10 d of monitoring. The silencing effect of DerL-2 KD fades after 5 d, and DerL-2 gene expression is restored to the same level as in WT after 8 d of growth. α-tubulin (αtub) was used as a normalizer. c) A stable bleached phenotype was observed throughout the monitoring period with an exceptional incidence of green cells in DerL-1 KD. The transient bleached phenotype of DerL-2 KD cells correlates with the renewal of gene expression, concomitant with the culture turning green again after 8 d of growth. d) Protein expression of selected chloroplast markers in DerL-1 and DerL-2 KDs; RuBisCO Activase (RCA) and ATP synthase β subunit (β-ATPase), chloroplast (shown with arrow) and mitochondrial homolog. Both chloroplast proteins display a drastic reduction of expression in DerL-1 KD by Western blot analysis compared with WT. Protein expression of RCA in DerL-2 KD gradually increases to the same level as in WT, and chloroplast β-ATPase expression is detectable from day 7. αtub expression was used as a control. Density measurements of Western blot analysis of chloroplast markers are provided in Table S1, and full gel images are available in the online repository (see Data availability).

To understand the function of the chloroplast-localized paralogs DerL-1 and DerL-2, the expression of these genes was downregulated individually by RNAi, and the cell growth, chloroplast morphology, and protein expression levels were compared to those observed in wild-type (WT) cells. RT-PCR (Nakazawa et al. 2015) showed that the silencing of DerL-1 persisted throughout the monitoring period of 10 d after the dsRNA electroporation (Fig. 2b). This silencing led to a notable delay in growth (Fig. 2a) and a stable bleached phenotype (Fig. 2c). Most knocked-down cells displayed chlorotic plastids when observed under the light microscope and were filled with numerous paramylon granules. Selected chloroplast protein markers, namely nucleus-encoded RuBisCO activase (RCA) and the chloroplast-encoded β-subunit of ATP synthase (β-ATPase), exhibited reduced expression (Fig. 2d, Table S1). The signal of RCA was almost undetectable throughout the entire monitoring period, and only the larger mitochondrial β-ATPase (56.5 kDa), not the chloroplastic β-ATPase (52.1 kDa), was expressed, unlike in the WT.

The silencing effect of DerL-2 diminished after day 5 after electroporation, with the level of its expression restored to the WT level (Fig. 2b, Table S1). Concomitantly, only a transient bleached phenotype was observed in this cell line, and a notable increase in the number of green cells was observed after day 7 (Fig. 2c). RCA expression was lower during the initial days of the monitoring period, coinciding with DerL-2 silencing (Fig. 2d). However, it began to increase from day 4 and appeared to be completely restored by day 7 (Table S1). Similarly, the signal for chloroplastic β-ATPase became detectable by day 7 (Fig. 2d, Table S1).

### Silencing of DerL-1 and DerL-2 leads to the downregulation of many chloroplast proteins

**Figure 3:**
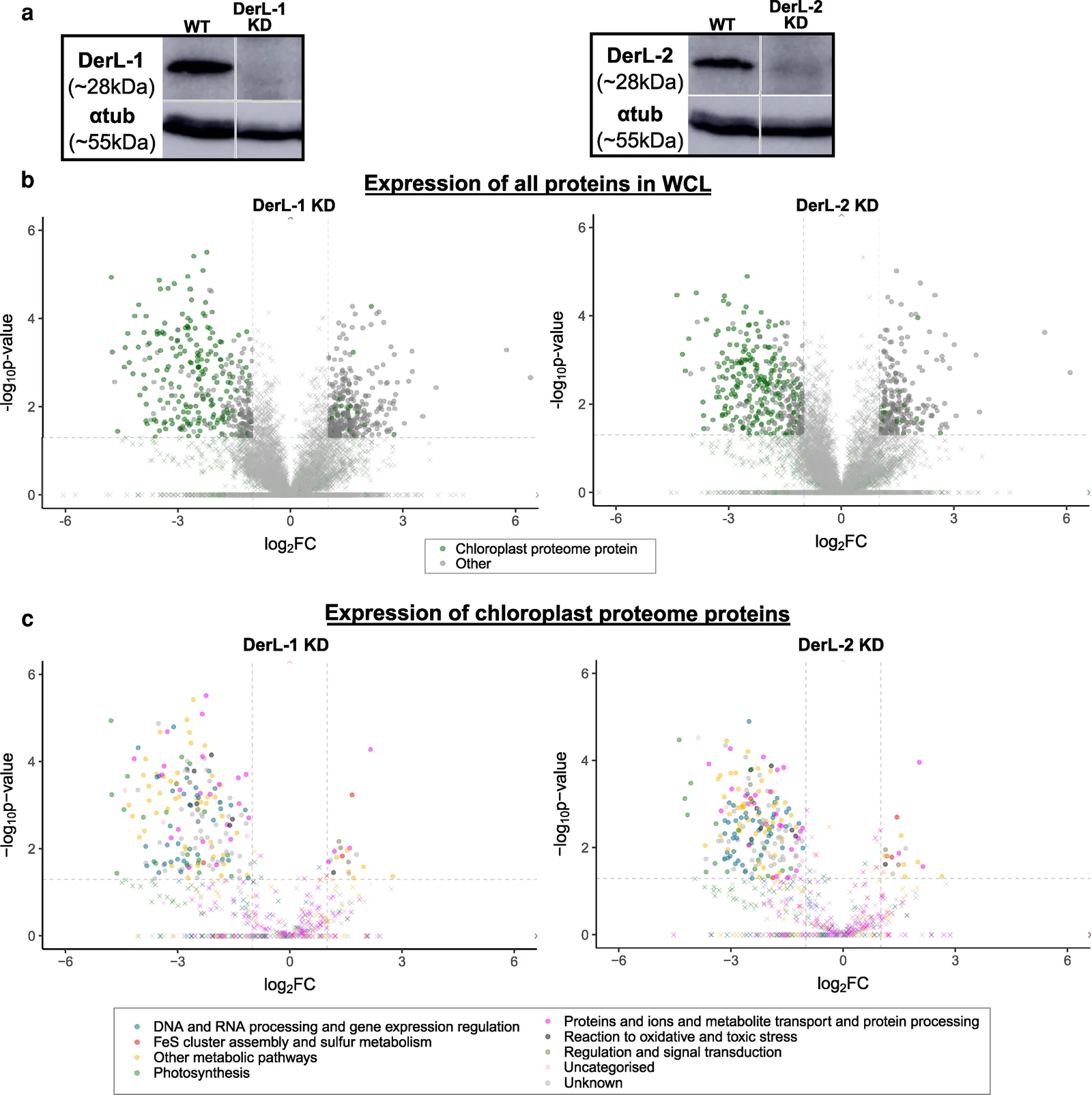
**Silencing of DerL-1 and DerL-2 lowers the expression of most chloroplast proteins on day 5 of growth.** a) Expression of DerL-1 and DerL-2 proteins is reduced on day 5 of RNA interference. ɑtub expression was used as a loading control. b) Volcano plots illustrating differential protein expression of DerL-1 KD and DerL-2 KD *E. gracilis* cells compared to WT on day 5 of RNA interference. Most chloroplast proteome proteins were downregulated in both DerL-1 and DerL-2 KDs. The threshold was set as ±1 of log_2_FC (fold change) and -log_10_(0.05) for *P*-value; non-significant changes are indicated as crosses. c) Same as b, but only chloroplast proteome proteins are displayed and color-coded according to functional categories. See Tables S2 and S3 for source data.

The changes in protein expression in DerL-1 and DerL-2 KD cells compared to the WT were assessed by differential proteomics in biological triplicates on day 5 after electroporation, a time when expression of both DerLs was undetectable (Fig. 3a). In total, we detected 9,226 proteins in whole cell lysates of which 596, 88, and 97 were unique to WT, DerL-1 KD, and DerL-2 KD respectively (Table S2, S3). 847 proteins were identified of the chloroplast proteome reported by Novák Vanclová et al. (2020), representing a 63% recovery of that proteome, which counts 1,345 proteins in total. This published set of 1,345 proteins will be referred to hereafter as the chloroplast proteome proteins.

DerL-1 KD led to the downregulation of 1,048 proteins and the upregulation of 973 proteins, using a fold change threshold of 2 × applied to all comparisons in this study. The expression of 5,660 proteins remained unchanged. Similarly, DerL-2 KD led to downregulation of 991 proteins and upregulation of 908 proteins; 5,924 proteins exhibited no change (Table S2, S3). Overall, the differential expression analysis of DerL-1 and DerL-2 KDs indicated a downregulation of the chloroplast proteome, with fewer chloroplast proteome proteins being upregulated (Fig. 3b). Specifically, in DerL-1 KDs, 332 chloroplast proteome proteins were downregulated, representing 50.0% of those for which the fold change could be determined. Upregulation was observed in only 64 (9.6%) chloroplast proteome proteins with fold changes. For DerL-2 KDs, 352 (50.1%) chloroplast proteome proteins were downregulated and 68 (9.7%) upregulated. Additionally, 123 (14.5%) chloroplast proteome proteins were detected solely in WT cells, while only 1 (0.1%) was found either only in DerL-1 or only in DerL-2 KDs and not in WT cells (Table S2, S3).

Sequence analyses indicate that many of the upregulated chloroplast proteome proteins exhibit non-canonical localization pre-sequences, or a lack thereof (Figure S1). There were 53 proteins upregulated in both DerL-1 and DerL-2 KD lines. Twenty-eight lack an identifiable SP, and 5 of these instead appear to encode a mitochondrial TP (Table S4). Twenty-four seem to carry multipartite localization signals, though confidence varies as to whether a TP follows the SP. For one (close homolog of glutathione S-transferase, EUGR57357), it could not be ascertained whether the first transmembrane domain represents a targeting (SP) or a functional domain. Given the challenges of identifying clear Class I or II chloroplast-targeting pre-sequences in the upregulated portion of the chloroplast proteome identified by Novák Vanclová et al. (2020), we propose that chloroplast-targeted proteins exhibit finely tuned sub-chloroplast localization and are not uniformly affected by DerL KD. Additional comments are provided in Table S4.

The chloroplast proteome proteins were classified into 7 functional categories, similarly to Novák Vanclová et al. (2020), but combining some categories to facilitate visualization (Table S3). Proteins that did not fit into any of these categories were labeled as uncategorized, while proteins lacking annotation were labeled as unknown. Every functional category displayed downregulation in both KDs (Fig. 3c, Table S3). The most affected category was photosynthesis, where all but 1 protein were either downregulated (41/57) or below the limit of detection (15/57) in DerL-1 KD. A similar trend was observed in the DerL-2 KD, where all but 2 proteins were either downregulated (40/57) or undetected (15/57) (Table S2, S3). Additionally, downregulation was noted in most proteins belonging to “other metabolic pathways”, 62/111 in DerL-1 KD and 67/111 in DerL-2 KD, and “DNA and RNA processing, gene expression regulation”, 57/104 in DerL-1 KD and 61/104 in DerL-2 KD. From the category “protein, metabolite, and ion transport and protein processing”, 49/171 and 51/171 were downregulated in DerL-1 KD and DerL-2 KD, respectively. In the category “reaction to oxidative and toxic stress”, 7/13 and 8/13 were downregulated in DerL-1 KD and DerL-2 KD, respectively, and 2/13 were upregulated in both. The last functional group, “regulation and signal transduction”, consisted of 23 proteins, with only 3 and 5 proteins downregulated in DerL-1 and DerL-2, respectively, and 3 proteins upregulated in both.

**Figure 4:**
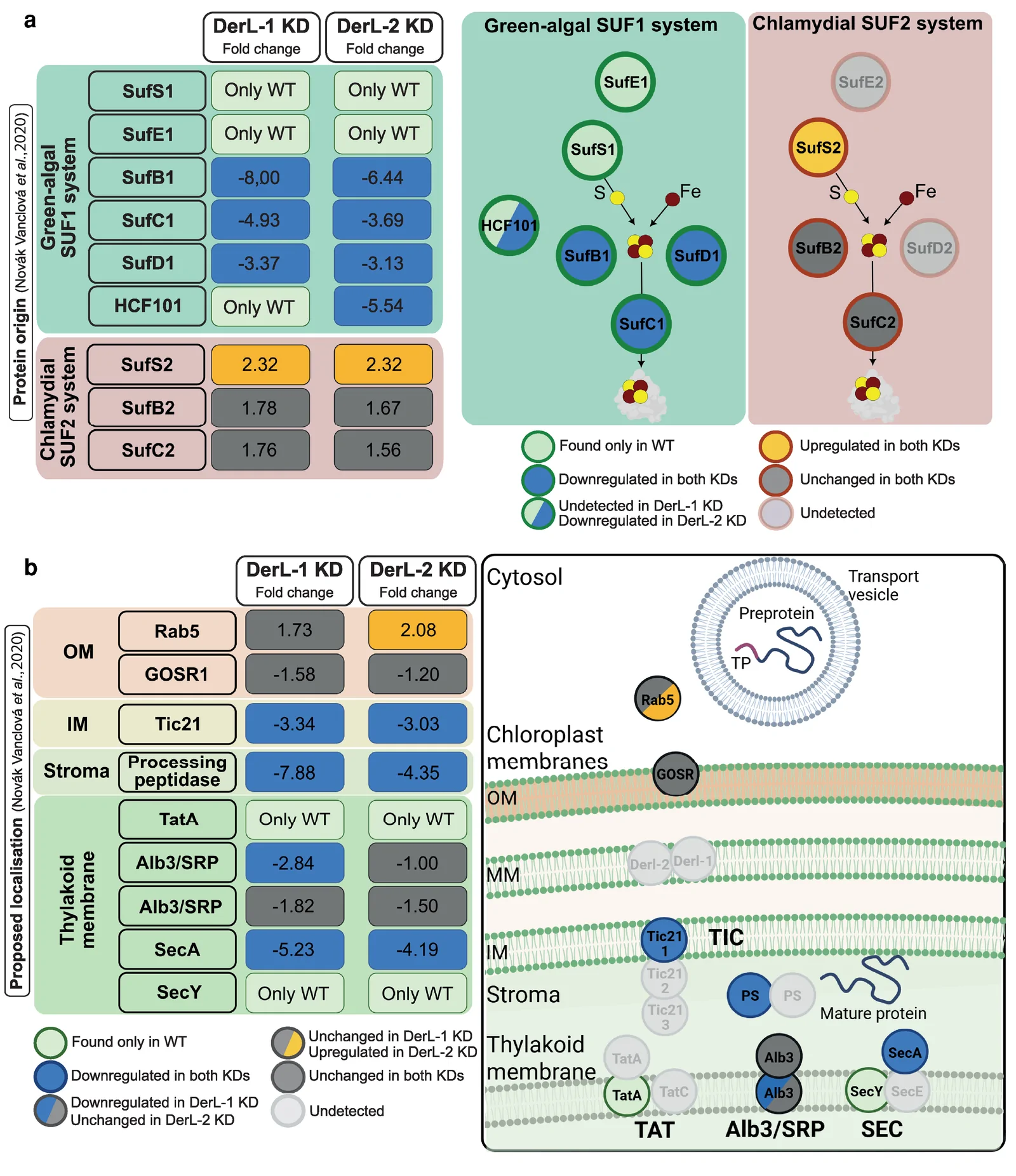
**Differential expression of proteins involved in 2 chloroplast systems after silencing of DerL-1 and DerL-2.** a) FeS cluster assembly SUF pathways. The SUF1 proteins phylogenetically related to green algae were downregulated or undetected in KDs. Similarly, the chloroplast FeS cluster carrier protein HCF101 was downregulated in DerL-2 KD and undetected in DerL-1 KD. In contrast, the expression of the other set of SUF2 proteins related to Chlamydiae was either unchanged or increased. Other proteins of this set were not detected. b) Proteins putatively involved in the chloroplast protein import machinery. Expression level of proteins predicted to the outermost membrane (OM)—Rab5 and GOSR1—remains unchanged or is upregulated. DerLs, proposed to be localized in the middle membrane (MM), were silenced by RNAi. Tic21, probably localized in the innermost membrane (IM), is downregulated, as is the processing peptidase (PS) in the stroma. Proteins in thylakoid membranes were mostly downregulated or undetected in DerL KDs. TP—targeting peptide. See Tables S5 and S6 for additional source data. The schemes were adapted from Novák Vanclová et al. (2020) and created in https://BioRender.com/cp3kb1l.

Exceptional was the situation with the proteins involved in FeS cluster assembly and sulfur metabolism. *E. gracilis* encodes 2 parallel SUF FeS cluster assembly pathways (Novák Vanclová et al. 2020). One of these is phylogenetically related to green algae (SUF1), while the other is related to Chlamydiae (SUF2), and they consist of 6 and 5 proteins, respectively (Table S5). Not all SUF proteins were detected in the published chloroplast proteome (Novák Vanclová et al. 2020), so we decided to have a closer look at their expression changes in our data. Of the green-algal SUF1 pathway, 3 and 4 proteins were downregulated in DerL-1 and DerL-2 KD, respectively. In addition, 3 and 2 were completely undetected in KDs, whereas in WT, they were quantifiable. Three proteins of the chlamydial SUF2 pathway were detected, of which 1 (SufS2) was upregulated in both KDs, while expression of the other 2 did not change (Table S5, Fig. 4a). Our observations strongly suggest that silencing of DerL proteins affects the 2 SUF pathways differently.

Finally, we identified 9 of the 18 proteins hypothesized to function in chloroplast protein import in *E. gracilis* (Novák Vanclová et al. 2020; Fig. 4b, Table S6). These proteins include Rab5, GOSR1, 1 paralog of Tic21, stromal processing peptidase, TatA, SecA, SecY, and 2 paralogs of Alb3. The expression of 2 proteins, which are proposed to localize or associate with the outermost membrane of the chloroplast, Rab5 and GOSR1, remained unchanged in DerL-1 KD. GOSR1 was also unchanged in DerL-2 KD, but Rab5 was slightly upregulated (2.08×). In contrast, the other proteins, expected to localize in the innermost envelope, thylakoid membranes, or in the chloroplast stroma, were either downregulated (Tic21, stromal processing peptidase, SecA) or undetected (TatA and SecY) in KD samples. The exception was Alb3, of which 1 paralog was downregulated in DerL-1 KD, while the second remained unchanged. Both paralogs remained unchanged in DerL-2 KD. Our observations strongly suggest that changes in the DerL KD chloroplast proteome composition are consistent with the phenotypic bleaching, particularly affecting stromal and thylakoid chloroplast proteins.

To see the effect of KDs on the localization of chloroplast proteome proteins, we have established the proteomes of the cytosol and the large granular fraction (LGF) containing organelles, including chloroplasts (Table S7, S8). The differential proteomics of KDs compared to WT showed clear trends of decrease in the chloroplast proteome proteins in both fractions (Table S9 and Figure S2). The changes in the abundances of proteins involved in FeS cluster synthesis and chloroplast protein import mostly agreed with observations from WCL analyses, except for the chlamydial SUF2 pathway in the cytosolic fraction, in which they were undetected. Meanwhile, the cytosolic fraction unexpectedly showed a high abundance of thylakoid SecA translocon protein (Tables S5, S6). These measurements do not indicate that silencing DerL-1 and DerL-2 proteins leads to the transfer of chloroplast proteins between fractions.

### Silencing of DerL-1 and DerL-2 causes morphological reduction of chloroplasts

**Figure 5:**
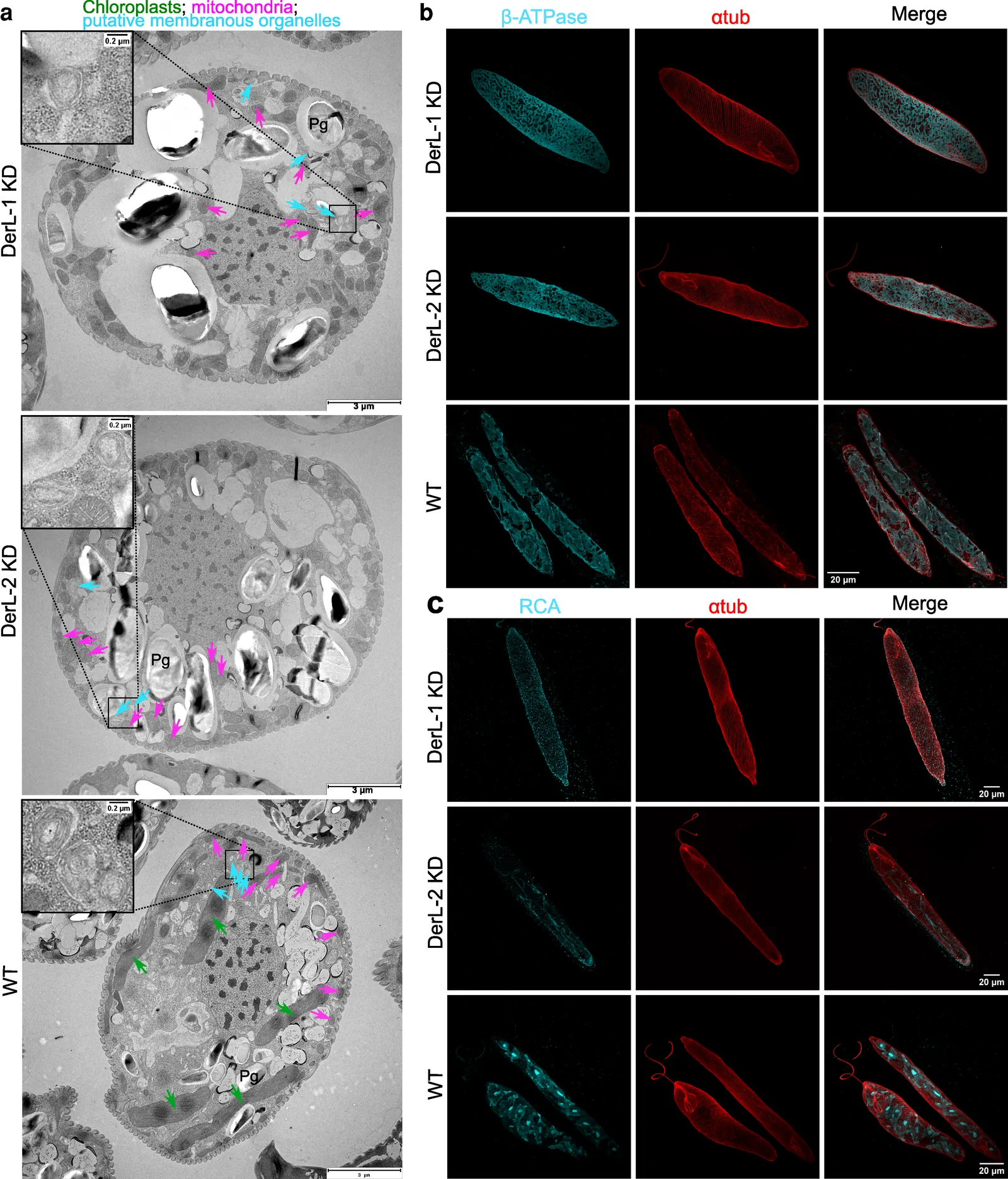
**Silencing of DerL-1 and DerL-2 causes a reduction of typical *E. gracilis* chloroplasts at the ultrastructural level on day 5 of growth.** a) Selected TEM of DerL-1, DerL-2 KD cells, and WT on day 5 of growth; chloroplasts, mitochondria, and membranous organelles are indicated with arrows. b) Selected micrographs from expansion microscopy of DerL-1 and DerL-2 KDs and WT on day 5 of growth, stained with rabbit anti-β-ATPase antibody and Guinea pig anti-αtub antibody. The scale bar is applicable to all images. c) Selected micrographs from expansion microscopy of DerL-1 and DerL-2 KDs and WT on day 5 of growth, stained with rabbit anti-RCA antibody and Guinea pig anti-αtub antibody. The scale bars are applicable to all images in the same row. See Videos 1 and 2 for 3D animated comparisons of these cells.

The silencing of DerL-1 and DerL-2 led to the loss of typical chloroplasts. On day 5, the morphology of organelles was evaluated using transmission electron microscopy (TEM) micrographs (Fig. 5a) and expansion microscopy (ExM) with anti-β-ATPase (Fig. 5b) and anti-RCA antibodies (Fig. 5c). The TEM micrographs of the WT cells showed large chloroplasts containing thylakoids (indicated by green arrows), a mitochondrion reticulum located under the cell surface (shown by magenta arrows), and numerous small paramylon granules (Fig. 5a). In DerL-1 KDs, multiple cross-sections of mitochondria were dispersed throughout the cell, while typical chloroplasts were absent. Large paramylon granules occupied most of the cell space, resulting in holes in the ultrathin sections. The phenotype of DerL-2 KDs was similar; however, the mitochondria were primarily located below the cell surface. In all cultures, the cells appeared to contain membranous organelles (indicated by cyan arrows) that we could not clearly ascribe to any known organelles and may represent highly underdeveloped chloroplasts (proplastids).

ExM of the WT cells showed the signal of β-ATPase antibody in both mitochondria and chloroplasts (Fig. 5b, Video 1), and the signal of RCA in chloroplasts, where it was particularly concentrated in pyrenoids, manifested as bright spots (Fig. 5c, Video 2). In contrast, no chloroplasts were detected in the DerL-1 KD, and only a nonspecific signal was observed in the pellicle. In DerL-2 KDs, however, the RCA signal was visible in a few small chloroplasts within some of the examined cells (Fig. 5c, Video 2). In both KD cell lines, β-ATPase displayed a dense network of thin threads forming a single mitochondrial reticulum that spanned the entire cell, primarily covering the sub-pellicular regions, consistent with TEM observations (Fig. 5b, Video 1).

## Discussion

This study provides evidence of the role of rhomboid pseudoproteases in chloroplast biogenesis of *E. gracilis*. The updated phylogenetic analysis with references from previous studies (Urban and Dickey 2011; Novák Vanclová et al. 2020; Karnkowska et al. 2023) revealed that euglenids encode 3 paralogs of these proteins (DerL-1-3), which form a robust clade sister to bacterial and archaeal proteins, and are distant from other eukaryotic homologs, including those of the SELMA translocon of complex plastids (Fig. 1a). AlphaFold predicted structures composed of 6 transmembrane domains, similar to the simplest form of rhomboid proteases found in bacteria (*E. coli* GlpG; Fig. 1d) (Urban and Dickey 2011). A key sequential distinction of euglenid DerLs from rhomboid proteases lies in the absence of the characteristic Ser-His dyad in the proteolytic pocket (Fig. 1c), which is essential for the proteolytic activity (Urban and Dickey 2011). While the conserved active-site structure is present, substitutions at the Ser and His sites with non-polar or hydroxylated residues suggest that euglenid DerLs have lost the proteolytic function. Such loss hints at alternative roles for euglenid DerL proteins, potentially related to protein translocation, signaling, or trafficking control as generally observed in other inactive rhomboid pseudoproteases, including Derlin proteins in ERAD and SELMA (Lemberg and Adrain 2016).

The presence of this clade of proteins exclusively in Euglenida (Lax et al. 2021) indicates that the common ancestor of this group likely acquired the ancestral prokaryotic DerL gene via horizontal gene transfer. This gene then diversified into 3 paralogs. The paralogue DerL-3 has the broadest phylogenetic distribution and may be functionally closest to the ancestral form. It is present in both heterotrophic and phototrophic species as well as the mixotroph *R. viridis*, which contains green algal kleptoplasts (Karnkowska et al. 2023). Though the localization of this protein has not been experimentally investigated, the presence of a mitochondrial targeting peptide implies a role in that organelle, warranting further investigation into its function. The presence of the DerL-1 and DerL-2 paralogs correlate with the presence of permanent secondary chloroplasts, indicating that their origin may be linked to the endosymbiosis of these organelles. Additionally, the presence of N-terminal multipartite targeting pre-sequences (Fig. 1b) and their enrichment in the chloroplast fraction (Novák Vanclová et al. 2020) strongly suggest their chloroplastidial functions, which were experimentally confirmed in this study.

Knocking down DerL-1 and DerL-2 individually resulted in a growth delay and a bleached phenotype that persisted for as long as the gene silencing was effective (see Fig. 2). This silencing persisted for 5 d in DerL-2, whereas DerL-1 expression did not fully restore during the 10-day monitoring period. These results are indicative of a similarly pivotal function for both proteins. Findings with similar regard were observed in *T. gondii*, where disrupting the expression of apicoplast Derlin also led to a growth delay, and this delay was reversed upon reintroduction of the Derlin protein (Agrawal et al. 2009). To our knowledge, there is no other report on the examination of Derlin functions in complex plastids.

Electron microscopy revealed the absence of chloroplasts with typical morphology in DerL KD cells. The cells also exhibited an expansion of the mitochondrial network throughout the cell (Fig. 5a and b), suggesting their reliance on heterotrophic growth. Unclassified, small, multimembrane organelles were observed in many cells from all 3 samples, DerL-1 and DerL-2 KDs, and WT, and we speculate that they may represent underdeveloped forms of plastids. These organelles, however, clearly differed in appearance from proplastids reported in dark-grown and UV- or hydrostatic pressure-bleached *E. gracilis* cells shown in Kivic and Vesk (1974), which displayed distinguishable triple membrane envelopes and clear thylakoid membranes inside. Additionally, they also differed from the double membrane-bound bodies, presumed to be thylakoid-devoid proplastids, which were described in *E. longa* (Webster et al. 1967). The membranous organelles reported here exhibited membranes loosely and evenly arranged throughout the structure, in contrast to the clearly separated thylakoids from the compact envelopes described by Kivic and Vesk (1974). They resemble the “concentric lamellae” bodies described in light-grown, streptomycin- and heat-bleached *E. gracilis* cells, interpreted as chloroplasts with deranged formation (Siegesmund et al. 1962). It should also be noted that these organelles were found in WT as well, so their presence is not strictly linked to the silencing of DerLs. In contrast to our study, Siegesmund et al. (1962) did not detect “concentric lamellae” bodies in WT, except for dark-grown cells exposed to light at 10 °C. Although those vesicles may represent some developmental stages of chloroplasts, we cannot exclude the possibility that they do not represent chloroplasts at all.

The most striking observation was the notable downregulation of organelle protein markers in DerL KDs. The anti-β-ATPase antibody detected only the mitochondrial paralogue on the Western blots from the DerL KDs (Fig. 2d), concomitant with the downregulation of the chloroplast paralogue (EUGR01798) and unchanged expression of the mitochondrial paralogue (EUGR30680) in both DerL KDs shown in the proteomic analysis (Table S2). Moreover, only a mitochondrial reticulum was observed by expansion microscopy (Fig. 5b). This reticulum consisted of numerous thin threads throughout the entire cell, resembling patterns seen in etiolated cells grown in the dark (Pellegrini 1980) or in cells from 3-day-old cultures (Konupková, Peña-Diaz, and Hampl 2025) maintained under the same photoheterotrophic conditions as in this study. Additionally, the lack of expression of RuBisCO activase (RCA, Fig. 2d), a nucleus-encoded protein, further supports the impairment of chloroplast function, which loses the ability to regenerate active RuBisCO and thereby fix CO_2_. RCA (EUGR24903) was downregulated in proteomic analysis of both DerL KDs, and its reduced expression was also observed by the expansion microscopy (Fig. 5c).

Comparative proteomics of DerL-1 and DerL-2 KDs relative to WT cells revealed a notable decrease in chloroplast protein expression. Specifically, 50% of the proteins identified in the chloroplast proteome (Novák Vanclová et al. 2020), with determined fold change, were downregulated in both DerL-1 and DerL-2 KDs (Fig. 3b, Table S2). Consistently, this was accompanied by bleaching and hampered culture growth even in the presence of organic carbon sources (Fig. 2a). Comparative proteomics of LGF and cytosolic fractions demonstrated the same trends, indicating that chloroplast proteome proteins do not accumulate in the cytosol (Figure S2).

The similar pattern of expression changes between DerL-1 and DerL-2 KDs further indicates their likely redundant or co-functional roles in the chloroplasts, where both are required together for proper function. In proteins putatively involved in protein import machinery, we observed a correlation between changes in protein expression and their proposed localization (Fig. 4b). Specifically, GOSR1 and Rab5 GTPase are hypothesized to occupy the outermost chloroplast membrane, involved in the docking of vesicles transporting proteins from the Golgi apparatus (Novák Vanclová et al. 2020). Notably, the expression of both proteins in both KDs remained unchanged or upregulated, suggesting that their insertion in the outermost membrane was not affected by the silencing of DerL-1 and -2. In contrast, Tic21 is hypothesized to be localized in the innermost membrane, and, concomitantly, its expression was downregulated in the cultures with silenced DerLs. Additionally, the stromal processing peptidase and the transporters to the thylakoids, TatA, SecA, and SecY, were also downregulated or only detected in WTs. These observations support the hypothesis that the impairment occurs in protein transport across the middle membrane due to the silencing of DerL-1 and DerL-2.

Another instance of proteins responding unevenly to the DerL KDs is those involved in chloroplast FeS cluster assembly. *E. gracilis* chloroplast contains 2 full sets of FeS cluster assembly pathways of different origin, each composed of SufE, SufS, SufB, SufD, and SufC proteins (Novák Vanclová et al. 2020). The expression of SUF1 proteins phylogenetically related to green algae was downregulated (Fig. 4a, Table S5), and the same applies to green alga-related chloroplast FeS cluster carrier protein HCF101, which was downregulated in DerL-2 KD and undetected in DerL-1 KD. These observations indicate that these proteins localize in the chloroplast stroma or thylakoids. In contrast, the expression of the other set of SUF2 proteins related to Chlamydiae was either unchanged (SufB2 and SufC2) or increased (SufS2). The remaining proteins of this set were not detected. One possible explanation for this observation is that these proteins do not need to be imported via DerL proteins, due to their localization in the intermembrane spaces of chloroplasts as proposed in Novák Vanclová et al. (2020). Targeted experiments and localizations are necessary to confirm and explain the contradictory changes exhibited by the 2 SUF systems.

For yet other KD-upregulated chloroplast proteome proteins, it is reasonable to propose that the observed upregulation may be compensatory—a physiological response to the failure to develop a photosynthetic chloroplast—or may reflect the ability of these proteins to localize to alternative cellular (sub)compartments, thereby avoiding downregulation caused by mislocalization. For example, *E. gracilis* possesses 4 closely related triose-phosphate isomerase (TPI) homologs—2 cytosolic and 2 plastid-localized (Figure S1; Füssy et al. 2020). All of them are retained in the non-photosynthetic sibling species *E. longa*, suggesting photosynthesis-independent functions they could facilitate in the heterotrophic DerL-KD cell lines as well. The upregulation of TPI in DerL-KD could constitute a metabolic shift to supply NAD(P)H to a malfunctioning plastid by providing substrate for the dark metabolism-induced glyceraldehyde-3-phosphate dehydrogenase (GapN, Füssy et al. 2020). Other upregulated proteins, eg, aldehyde dehydrogenase (EUGR55571), which do not show multipartite targeting pre-sequences (Figure S1), may be tethered to the outer membrane by a transmembrane domain, or docked through protein–protein interactions, and thus remain largely unaffected by DerL knockdown.

Our data clearly demonstrate that DerL-1 and DerL-2 are essential for the biogenesis of chloroplasts in *E. gracilis*. Since these proteins are membrane proteins, and their distant homologs in the ERAD and SELMA pathways have been shown to facilitate protein translocation across membranes, we speculate that a similar role may apply to DerL-1 and DerL-2 in the chloroplasts of *E. gracilis*. Specifically, these proteins may serve as a functional replacement for the missing TOC complex and participate in protein import through the middle membrane of the secondary chloroplast in *E. gracilis*. This hypothesis provides a plausible explanation for the downregulation of a large part of the chloroplast proteome and morphological reduction of chloroplasts, while some proteins localized in the outer chloroplast membrane and in the intermembrane space remain expressed. To further corroborate this hypothesis, detailed localization studies—which we were unable to achieve with the available antibodies—and protein import experiments are necessary. If correct, the situation in *E. gracilis* would be reminiscent of the SELMA machinery in red-alga-derived complex plastids, which facilitate protein import across the second outermost membrane. Interestingly, the functional similarity of DerL-1 and DerL-2 to SELMA components does not align with their origin. DerLs in *E. gracilis* do not have specific relations to components of the SELMA system; instead, they belong to a euglenid-specific clade of rhomboid pseudoproteases independently arising from prokaryotes. Among these, 1 paralog has an unclear function, probably within the mitochondrion, while the 2 investigated in this study likely play a role in protein import to the chloroplast. If our hypothesis holds true, it will represent a fascinating example of convergence at the molecular level, illustrating how evolution can independently recruit proteins from the same family to address similar functional challenges in different biological contexts.

## Materials and methods

### Sequence and structure analysis and phylogeny

Reference sequences from previous studies (Urban and Dickey 2011; Novák Vanclová et al. 2020) were used to query the EukProt (Richter et al. 2022), EggNOG (Huerta-Cepas et al. 2019), and the *R. viridis* proteomes (Karnkowska et al. 2023). Up to 500 target sequences with e-value lower than 1e−3 were retrieved by DIAMOND v2.1.9.163 (Buchfink et al. 2015), then clustered using MMseqs2 release 14-67949d7 on 80% identity, over 80% coverage, clustering mode 2 (Steinegger and Söding 2017) while keeping the reference sequences, and manually pruned. The resulting sequences were aligned with Muscle5.1 (Edgar 2022), trimmed automatically to retain positions with <50% gaps with trimAl v1.2rev59 (Capella-Gutiérrez et al. 2009), and manually to remove noisy N-termini. Phylogenies were inferred with IQ-TREE v2.3.4 (Minh et al. 2020), with the EX3 model for hydrophobic proteins selected for the guide tree and the ELM + C60 + G (Eukaryotic Linked Mixture) model selected for the posterior mean site frequency analysis (Wang et al. 2018). Structural predictions were made with AlphaFold 3 Server (last accessed on 20 October 2024; Abramson et al. 2024), and the round 4 models were aligned with UCSF ChimeraX v1.8 Matchmaker (Meng et al. 2023). Additionally, *E. coli* GlpG protease (PDB:2NRF) was used for structural comparisons. Protein sequence alignment visualizations were made with Geneious Prime 2024.0.7 (Kearse et al. 2012). TM-scores were calculated by TM-align v20190822 (Zhang and Skolnick 2005) accessed online, and normalized by the longer chain length (DerL-1, 186 residues, when compared to DerL-2 and DerL-3; DerL-2, 184 residues, when compared to DerL-3, 178 residues).

Spliced leaders (SL; here represented by a shorter sequence tag TTTTTCG frequently found in *E. gracilis* transcript sequences) were identified using tblastn against published *E. gracilis* transcriptome shotgun assemblies (TSA), accessions GDJR01 (https://www.ncbi.nlm.nih.gov/nuccore/1000372679), GEFR01 (https://www.ncbi.nlm.nih.gov/nuccore/1127253110), and HBDM01 (https://www.ncbi.nlm.nih.gov/nuccore/2981516568). Protein models were corrected to truncate any amino acid residues preceding the first methionine that follows the SL sequence, ie the true ORF start. The accessions of cytosolic and plastid triose-phosphate isomerase homologs of *E. gracilis* and *E. longa* were obtained from (Füssy et al. 2020). Protein domains were determined using the InterProScan service of Geneious Prime v2025.2.2 against the PANTHER, NCBIfam, Phobius, SignalP, and TMHMM databases of HMM profiles (Kearse et al. 2012; Jones et al. 2014). Localization signals were predicted using PrediSi (Hiller et al. 2004), PredSL (Petsalaki et al. 2006), and MultiLoc2 (Blum et al. 2009) as in (Záhonová et al. 2018). SP was considered present if any of PrediSi, PredSL, or Phobius predicted the motif.

### Cell culture

*E. gracilis* Z strain was grown in Cramer-Myers media (CMM; Cramer and Myers 1952), pH 6.8, with 0.4% (v/v) ethanol (CMMEt) as a carbon source. The culture was maintained in 25 cm^2^ vented cap culture flasks in a Q-Cell 60 incubator (Pol-Lab, Wilkowice, Poland) at 25 °C, with 12 h light and 12 h dark settings.

### RNA interference

RNA interference (RNAi) was performed according to Nakazawa et al. (2015). Briefly, double-stranded RNA (dsRNA) was synthesized using the MegaScript RNAi Kit (Thermo Fisher Scientific, Waltham, MA, United States) with 1 μg of template DNA, specifically prepared for each gene of interest using primers with additional T7 promoter sequences (Table S10). The optimal binding site for primers amplifying the sequence containing motifs that can function as highly effective siRNAs was determined based on the siRNA prediction tool (http://sidirect2.rnai.jp/). 15 μg of dsRNA (or 15 μL of elution buffer from the MegaScript RNAi Kit for the negative control) were electroporated into 400 μL of 1 × 10^7^ cells/mL suspension in PBS with 0.5 mM Mg(OAc)_2_ and 0.09 mM Ca(OAc)_2_ in a 0.2 cm cuvette by Gene Pulser Xcell Electroporation Systems (Bio-Rad Laboratories, Hercules, CA, United States) with the same setting as in Nakazawa et al. (2015). Cells were transferred to fresh CMMEt and recovered in the dark for 4 h.

### Growth curve measurements

Growth curve measurements were prepared in biological triplicate. For DerL-1 knockdown (KD), DerL-2 KD, and the negative control (WT), 25 cm^2^ culture flasks containing 20 mL of media were inoculated with 10^6^ cells on Day 0, and cells were counted daily at the beginning of the light phase for 10 consecutive days. Cell counts were acquired with a Beckman Coulter Z2 Cell and Particle Counter (Beckman Coulter, Brea, CA, United States). Each sample was counted in a technical triplicate, and the means with standard deviation were calculated using RStudio version 4.3.3 (Posit Team 2024, Posit Software, PBC, Boston, MA, United States).

#### Reverse transcription PCR (RT-PCR)

From day 3, when cultures reached sufficient density, cells were also collected for RNA isolation. RNA isolated by TRI Reagent (Invitrogen, Thermo Fisher Scientific, Waltham, MA, United States) was reverse transcribed with PrimeScript RT Master Mix (Takara Bio, Tokyo, Japan) to confirm gene silencing by RT-PCR as described in Nakazawa et al. (2015). Pairs of primers were designed for detection (Table S10) and used for PCR with EmeraldAmp GT PCR Master Mix (Takara Bio) and cDNA template according to the manufacturer's instructions for 32 cycles for DerL-1, 30 cycles for DerL-2, and 18 cycles for α-tubulin, which was used as a normalizer.

#### Immunodetection of protein markers expression

From day 3, when cultures reached sufficient density, cells were also collected for immunoblot analyses of chloroplast protein markers. DerL-1 KD, DerL-2 KD, and WT whole cell lysates were prepared as follows: 1 × 10^6^ cells were pelleted at 1,000 × *g* and resuspended in 100 μL of Laemmli buffer, boiled at 95 °C for 5 min, of which 15 μL were loaded and run in 12% SDS-PAGE gels followed by immunoblot analysis on PVDF membranes. 5% (w/v) low-fat milk suspension in PBS + 0.1% (v/v) Tween (PBS-T) was used as a blocking buffer. Antibodies against RuBisCO activase, raised in rabbit (anti-RCA; AS10 700, Agrisera, Vännäs, Sweden) and against ATP synthase β-subunit of *Trypanosoma brucei*, raised in rabbit (anti-β-ATPase; kindly provided by Alena Zíková, Biology Centre of the Czech Academy of Sciences, České Budějovice, Czech Republic), were diluted 1:5,000 and 1:10,000 in PBS-T, 5% (w/v) low-fat milk suspension, respectively, and used for the immunodetection of chloroplast marker proteins. As a loading control, α-tubulin was detected using anti-acetylated α-tubulin antibody raised in mouse (anti-αtub, ECACC 00020913) in a 1:5,000 dilution in PBS-T, 5% (w/v) low-fat milk suspension. Horseradish peroxidase-labeled secondary antibodies, goat anti-rabbit IgG (A9169) and goat anti-mouse IgG (AP308P) antibody (both Sigma-Aldrich, Darmstadt, Germany) were used for immunodetection on immunoblot by chemiluminescence with Clarity Max Western ECL Substrate (Bio-Rad Laboratories, CA, United States) on a GE Amersham Imager 600 Luminescence Image Analyzer (General Electric, Woonsocket, RI, United States). For the detection of DerL-1 and DerL-2 proteins, 2 × 10^6^ cells collected on day 5 of the RNA interference were solubilized in 100 µL of Laemmli buffer for SDS-PAGE and Western blot. Antibodies against DerL-1 and DerL-2 were custom-made as conjugates with horseradish peroxidase (Tresars LTD, London, United Kingdom) and were diluted 1:5,000 in TBS-T, 5% (w/v) BSA. TBS-T was also used for prolonged overnight washing of PVDF membranes.

### Proteomic analysis

Twenty mL of a 5-day-old culture was used for proteomic measurements. The culture was pelleted by centrifugation at 1,000 × *g*, 1 min, and the pellet was washed 2× with GRM buffer (50 mM HEPES-KOH pH 8, 330 mM sorbitol, 2 mM EDTA, 1 mM MgCl_2_, and 1 mM MnCl_2_; Napier and Barnes 1995). A third wash was performed with ice-cold buffer with additional cOmplete Mini EDTA-free protease inhibitor cocktail (Roche, Basel, Switzerland). The cells were very gently ruptured by French-press G-M Model 11 (GlenMills, Clifton, New Jersey, United States) with a cell pressure of 2,400 kPa at a low ratio. The unbroken cells were pelleted repeatedly at 250 × *g* until no unbroken cells were present in the supernatant. The unbroken cells were diluted in GRM buffer, ruptured twice at a higher cell pressure of 10,000 kPa at a low ratio, and stored at −80 °C for a whole cell lysate MS analysis. The supernatant containing only the organellar and cytosolic fractions was pelleted by centrifugation at 3,000 × *g,* and the pellet was collected as a LGF. The remaining sample was spun at 120,000 × *g,* and the supernatant was collected as a cytosolic fraction. Both fractions were stored in −80 °C for an MS analysis.

Proteomic quantification was performed by a proteomic facility at BIOCEV. The samples were lysed by boiling at 95 °C for 10 min in 100 mM TEAB (Triethylammonium bicarbonate) containing 2% (w/v) SDC (sodium deoxycholate), 40 mM chloroacetamide, 10 mM TCEP (Tris(2-carboxyethyl)phosphine), and further sonicated (Bandelin Sonoplus Mini 20, MS 1.5). Protein concentration was determined using the BCA protein assay kit (Sigma Aldrich, Darmstadt, Germany), and 10 µg of protein per sample was used for MS sample preparation. The sample volume was then adjusted to 65 µl by adding 100 mM TEAB containing 2% (w/v) SDC. Samples were further processed using SP3 beads with the Thermo KingFisher Flex automated Extraction & Purification System in a 96-well plate. Briefly, 65 µl of the sample was added to 65 µl of 100% ethanol and mixed with the SP3 beads. After binding, the beads were washed 3 times with 80% ethanol. After washing, samples were digested in 50 mM TEAB at 40 °C with 1 µg of trypsin for 2 h, then another 1 µg of trypsin was added and digested overnight. After digestion, samples were acidified with 1% (v/v) trifluoroacetic acid to a final concentration, and peptides were desalted using in-house-made stage tips packed with C18 disks AttractSPE (Affinisep, Le Houlme, France) according to Rappsilber et al. (2007).

Nano Reversed phase columns (Ion Opticks, Aurora Ultimate TS 25 × 75 C18 UHPLC column) were used for LC/MS analysis. Mobile phase buffer A was composed of water and 0.1% (v/v) formic acid. Mobile phase B was composed of acetonitrile and 0.1% formic acid. Samples were loaded onto the trap column (C18 PepMap100, 5 μm particle size, 300 μm × 5 mm, Thermo Scientific) for 4 min at 18 μL/min loading buffer was composed of water, 2% (v/v) acetonitrile and 0.1% (v/v) trifluoroacetic acid. Peptides were eluted with Mobile phase B gradient from 4% to 35% B in 60 min. Eluting peptide cations were converted to gas-phase ions by electrospray ionization and analyzed on a Thermo Orbitrap Ascend (Thermo Scientific) by data-independent approach. Survey scans of peptide precursors from 350 to 1,400 m/z were performed in Orbitrap at 60 K resolution (at 200 m/z) with a 4 × 10^5^ ion count target. DIA scans were performed in Orbitrap at 30 K resolution. AGC target was set to 1,000% and the maximum injection time mode was set to Auto. Precursor mass range 400 to 1,000 m/z was covered by 30 windows 20 Da wide. Activation type was set to HCD with 28% collision energy.

All data were analyzed and quantified with the Spectronaut 19 software (Bruderer et al. 2015) using directDIA analysis. Data were searched against the in-house *E. gracilis* database containing 68,573 entries, based on the published transcriptomes (O’Neill et al. 2015; Yoshida et al. 2016; Ebenezer et al. 2019) and provided in Table S11. Enzyme specificity was set as C-terminal to Arg and Lys, also allowing cleavage at proline bonds and a maximum of 2 missed cleavages. Carbamidomethylation of cysteines was set as a fixed modification, and N-terminal protein acetylation and methionine oxidation as variable modifications. Quantification was performed on the MS2 level. Precursor PEP cutoff and precursor and protein cutoff were set to 0.01, protein PEP was set to 0.05. Data were exported, and data analysis was performed using Perseus 1.6.15.0 (Tyanova et al. 2016). The volcano plots were prepared using RStudio version 4.3.3 (Posit Team 2024, Posit Software, PBC, Boston, MA, United States).

### Transmission electron microscopy

For ultrastructural analysis by TEM, 5-day-old cells grown on CMMEt were harvested and pelleted by centrifugation at 400 × *g* for 30 s. Cells were fixed in a solution of 2.5% (v/v) glutaraldehyde and 4% (v/v) formaldehyde in 0.1 M cacodylate buffer for 1 h, then washed and embedded in 2% (w/v) low-melting agarose as a cell pellet. Dissected pieces (1 × 1 × 1 mm) of agarose-embedded pellet were then post-fixed in osmium tetroxide [1% (w/v) in distilled water] for 1 h on ice, then by reduced osmium [1% (w/v) osmium tetroxide and 1.5% (w/v) ferrocyanide in distilled water] for 1 h on ice. After washing with distilled water, samples were dehydrated in 50% and 75% ethanol (10 min each), then subjected to en bloc staining in 0.5% (w/v) uranyl acetate in 75% ethanol overnight at 4 °C, followed by 3 washes in 75% ethanol. The samples were further dehydrated in 85%, 95%, and 100% ethanol (10 min each) and subsequently in propylene oxide (2 times for 20 min). Samples were then embedded in SPURR low viscosity embedding kit (EMS#14300) and cured at 70 °C for 12 h. The ultrathin 60 nm sections were cut with a diamond knife using LEICA EM UC7 ultramicrotome (Leica, Wetzlar, Germany), collected on Cu slot grids with 1% formvar and 2 nm carbon, and post-contrasted with 3% lead citrate for 2 min. Imaging data were acquired on a JEM 2100 Plus transmission electron microscope (JEOL, Tokyo, Japan) operated at 200 kV using a TVIPS XF 416 camera.

### Expansion microscopy

Expansion microscopy of DerL-1 and DerL-2 KD cells was performed as described in Konupková et al. (2025). Briefly, 5-day-old cells were collected, resuspended in fresh CMMEt, and fixed with 4% (v/v) formaldehyde and 4% (v/v) acrylamide. The suspension was transferred to a poly-L-lysine-coated round coverslip in a 24-well plate and left overnight. The next day, the coverslip was washed with PBS and transported to a drop of the monomer solution in a wet chamber on ice on parafilm, allowed to set for 5 min, and then moved to 37 °C for 30 min. The gel was detached from the coverslip in the denaturation buffer and then boiled for 1 h at 95 °C in the same buffer in a 1.5 mL tube. Next, the gel was washed 3 × in milliQ water in a Petri dish. Squares of approximately 1 × 1 cm were cut and placed into a 24-well plate to be blocked with 2% (w/v) bovine serum albumin (BSA) in PBS and incubated with primary antibodies at 4 °C overnight. The following primary antibodies were used: anti-acetylated-α-tubulin raised in guinea pig (anti-αtub; AA345, ABCD antibodies, Geneva, Switzerland), anti-β-ATPase raised in rabbit and kindly provided by Dr Zikova, and anti-RCA raised in rabbit (Agrisera, AS10 700), all diluted 1:300. The following day, samples were washed 3 × with PBS and secondary antibodies were applied. Alexa Fluor 488, goat anti-rabbit IgG (A-11008); Alexa Fluor 594, goat anti-guinea pig IgG (A-11076) (all Invitrogen, Waltham, MA, United States) were diluted 1:300 in 2% (w/v) BSA in PBS and incubated at 4 °C overnight. Finally, the samples were washed 2 × with PBS and 3 × with milliQ water to expand the gel. The expanded gel was transferred to a poly-L-lysine-coated 35 mm glass-bottom dish and observed under a spinning disk Nikon CSU-W1 (Nikon Instruments, Tokyo, Japan) microscope with a CF Plan Apo VC 60XC WI objective (with water immersion). A quad-band dichroic mirror 405/488/561/638 nm was used for laser excitation of wavelengths 488 and 561 nm for AlexaFluor 488 and 594, respectively. NIS-Elements (with AI) 6.0.2 software was used for capturing images with a PRIME BSI camera (Teledyne Photometrics, Tucson, AZ, United States). Images with anti-β-ATPase were deconvolved using SVI Huygens Professional 22.10 software (Scientific Volume Imaging, Hilversum, the Netherlands, http://svi.nl), and all images were post-processed as a maximal projection in Fiji open-source software 2.16.0/1.54p (Schindelin et al. 2012). The 3D models were prepared using Imaris 9.9.1. and merged in Zoner Photo Studio X (Zoner, Brno, Czech Republic) to make the Videos.

### Accession numbers

Sequence data from this article can be found in Table S11.

## Supplementary Material

kiag253_Supplementary_Data

## Data Availability

Alignments and source data are available on FigShare https://doi.org/10.6084/m9.figshare.30384367.v1. Interactive tree of the rhomboid family available on iTOL: https://itol.embl.de/tree/1602171292165061730081700. The mass spectrometry proteomics data have been deposited to the ProteomeXchange Consortium via the PRIDE (Perez-Riverol) partner repository with the dataset identifier PXD073723.
