# Supplementary material for: Derlin-like proteins are essential for the function of Euglena gracilis chloroplasts
Source: Plant Physiol. 2026 Apr 29;201(2):kiag253. doi: 10.1093/plphys/kiag253 (PMC13602068; doi:10.1093/plphys/kiag253)
Supplement: Supplementary file 1 [file kiag253_Supplementary_Data.zip › Supplementary Figures.pdf]

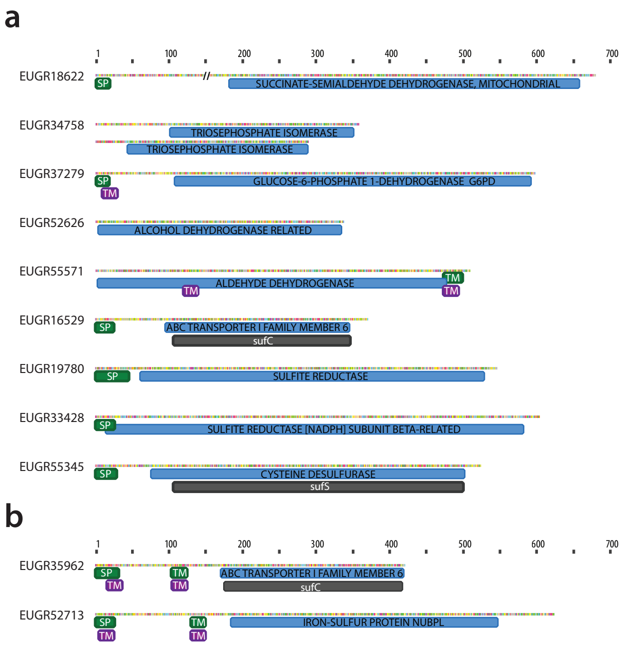

c

SL	PrediSi	PredSL	MultiLoc2
Y	–	SP	–
Y/Y	–/–	SP/–	mTP/nd
Y	SP	SP	cTP
Y	–	–	nd
Y	–	–	nd
Y*	SP	SP	cTP
Y	–	mTP	–
Y*	–	SP	cTP
Y	–	mTP	cTP
Y	SP	SP	cTP
Y	SP	SP	cTP

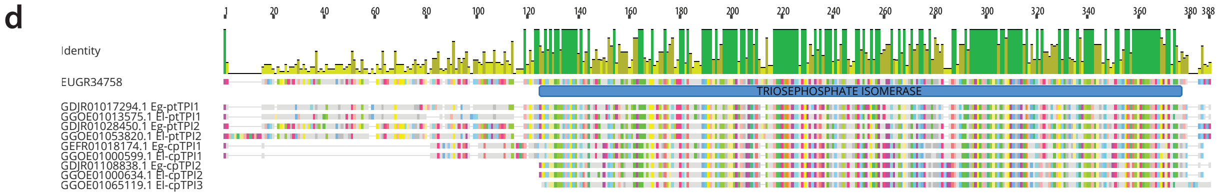

Fig. S1: Domain structure of selected putative chloroplast-targeted proteins of *E. gracilis*. a) Domain structure of upregulated proteins showing atypical N-terminal chloroplast-targeting signals, in scale (above the panel). Colored shapes denote regions with domain annotation as determined by PANTHER (blue), NCBIFAM (grey), Phobius (green), and TMHMM (purple) hidden Markov model searches; SP, signal peptide as predicted by PHOBIUS; TM, transmembrane domain as predicted by PHOBIUS or TMHMM. Two alternative protein models were considered for EUGR34758 with a region in the functional domain highly identical in sequence (3 substitutions over 166 aligned amino acids). // denotes an excised 700-amino acid segment of EUGR18622 that has no domain annotation; the total length of the protein model is 1,377 amino acids. **b)** In scale example domain structures of canonical plastid-targeted proteins (Class I multipartite targeting pre-sequences) of the iron-sulfur assembly pathway that were observed to be significantly downregulated or absent in DerL-KDs. **c)** Completeness of corresponding transcripts and localization predicted based on full-length proteins sequences. SL denotes whether spliced leaders were found at the beginning of each transcript. Asterisks mark the following notes: for EUGR16529, the transcript does not encode a methionine in-frame immediately after the SL sequence – the open reading frame (ORF) start was determined by comparison with *Euglena longa* transcript GGOE01061672.1; for EUGR33428, none of the corresponding transcripts had a SL, but there is a stop codon four codons upstream of the assumed ORF start, and a close complete homolog from *E. longa* with a SL with 88% sequence identity in the first 50 nucleotides appears to use the same ORF start position, corroborating this ORF. Columns PrediSi, PredSL, MultiLoc2, and DeepLoc 2.0 list the predicted signals in full length proteins by the respective algorithms. PrediSi was run with full-length sequences in Eukaryotic mode; PredSL was run with full-length sequences in non-plant mode; MultiLoc2 was run with trimmed sequences (signal peptides as determined by PredSL cleaved off) in plant low-resolution

mode, DeepLoc-2.0 was run with full-length sequences in high-quality mode. cTP, chloroplast transit peptide (note, in non-plant mode this would correspond to a long mitochondrial transit peptide, but DeepLoc-2.0 does not accept a lineage setting); mTP, mitochondrial transit peptide; SP, signal peptide; TM, transmembrane domain; nd, not determined (no signal to cleave); –, no targeting signal detected, i.e., assumed cytosolic localization. **d)** Multiple sequence alignment of triose-phosphate isomerases (TPI) in *E. gracilis* and *E. longa*. Labels consist of NCBI accession, species (Eg or El), localization (cp and pt for cytoplasm and plastid, respectively), and numbers indicating orthology between gene family members (e.g., Eg-cpTPI1 orthologous to El-cpTPI1). EUGR34758 is identical to Eg-ptTPI2.

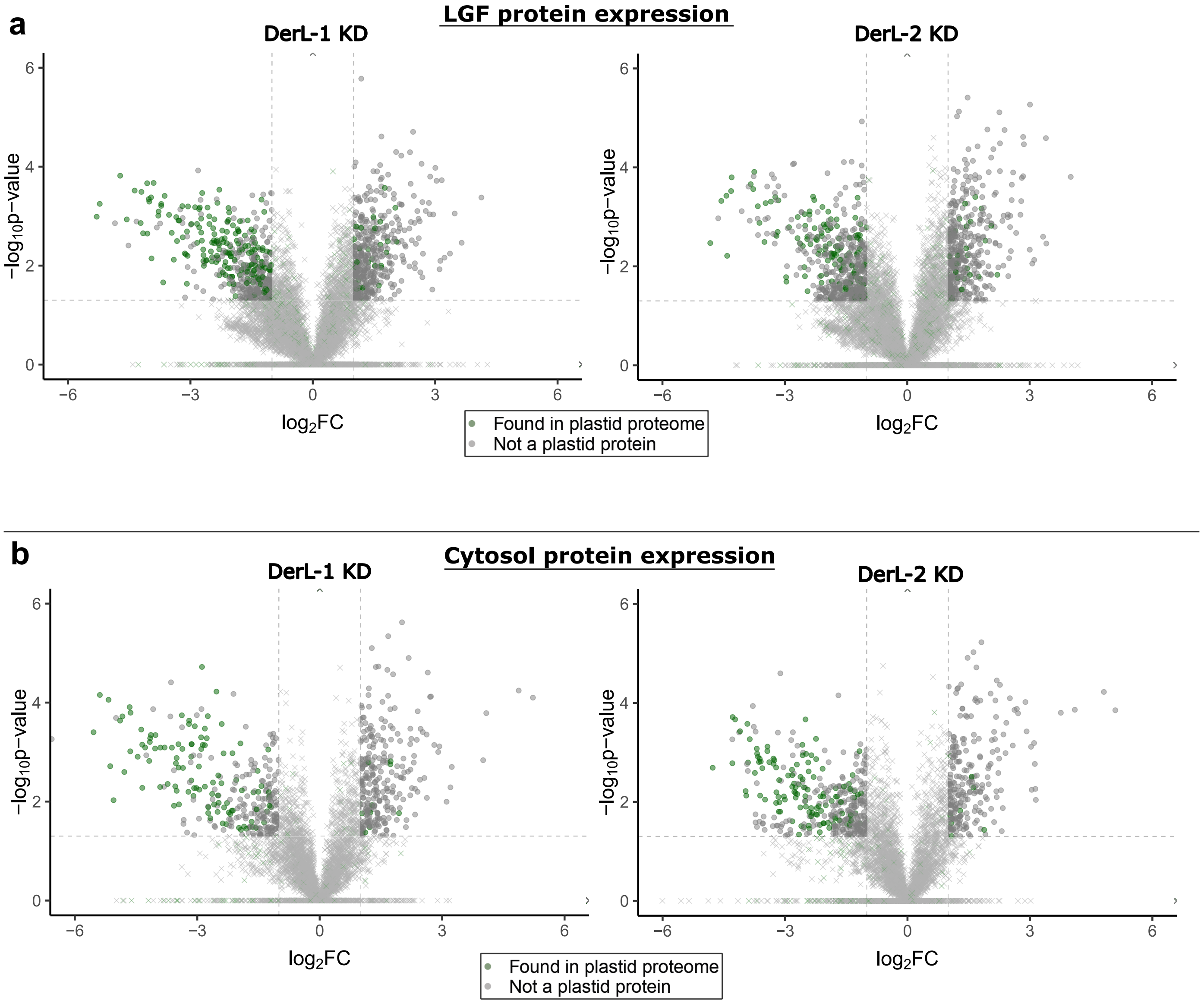

Fig. S2: Silencing of DerL-1 and DerL-2 lowers the expression of most chloroplast proteins in large granular fraction (LGF) and cytosol, where the chloroplast proteins are not accumulated. Volcano plots illustrating differential protein expression of DerL-1 KD and DerL-2 KD *E. gracilis* cells compared to WT on day 5 of RNA interference in a) LGF and b) cytosol. Most chloroplast proteome proteins (green) were downregulated in both DerL-1 and DerL-2 KDs in both fractions. The threshold was set as ±1 of log2FC (fold change) and -log10(0.05) for p-values; non-significant changes are indicated as crosses. See Tables S7 and S8 for source data
